# The Enhanced Cytotoxic Effects in B-Cell Leukemia and Lymphoma Following Activation of Prostaglandin EP4 Receptor and Targeting of CD20 Antigen by Monoclonal Antibodies

**DOI:** 10.3390/ijms23031599

**Published:** 2022-01-29

**Authors:** Tijana Markovič, Helena Podgornik, Damjan Avsec, Sanja Nabergoj, Irena Mlinarič-Raščan

**Affiliations:** 1 Faculty of Pharmacy, University of Ljubljana, SI-1000 Ljubljana, Slovenia; tijana.markovic@ffa.uni-lj.si (T.M.); helena.podgornik@kclj.si (H.P.); damjan.avsec@ffa.uni-lj.si (D.A.); sanja.nabergoj@ffa.uni-lj.si (S.N.); 2 Department of Haematology, University Medical Centre Ljubljana, SI-1000 Ljubljana, Slovenia

**Keywords:** B-cell leukemia and lymphoma, chronic lymphocytic leukemia, prostaglandin EP4 receptor, selective EP4 receptor agonist, monoclonal antibodies, synergistic effects

## Abstract

Anti-CD20 monoclonal antibodies (MAbs) have revolutionized the treatment of B-cell leukemia and lymphoma. However, many patients do not respond to such treatment due to either deficiency of the complementary immune response or resistance to apoptosis. Other currently available treatments are often inadequate or induce major side effects. Therefore, there is a constant need for improved therapies. The prostaglandin E2 receptor 4 (EP4) receptor has been identified as a promising therapeutic target for hematologic B-cell malignancies. Herein, we report that EP4 receptor agonists PgE1-OH and L-902688 have exhibited enhanced cytotoxicity when applied together with anti-CD20 MAbs rituximab, ofatumumab and obinutuzumab in vitro in Burkitt lymphoma cells Ramos, as well as in p53-deficient chronic lymphocytic leukemia (CLL) cells MEC-1. Moreover, the enhanced cytotoxic effects of EP4 receptor agonists and MAbs targeting CD20 have been identified ex vivo on primary lymphocytes B obtained from patients diagnosed with CLL. Incubation of cells with PgE1-OH and L-902688 preserved the expression of CD20 molecules, further confirming the anti-leukemic potential of EP4 receptor agonists in combination with anti-CD20 MAbs. Additionally, we demonstrated that the EP4 receptor agonist PgE-1-OH induced apoptosis and inhibited proliferation via the EP4 receptor triggering in CLL. This work has revealed very important findings leading towards the elucidation of the anticancer potential of PgE1-OH and L-902688, either alone or in combination with MAbs. This may contribute to the development of potential therapeutic alternatives for patients with B-cell malignancies.

## 1. Introduction

B-cell malignancies represent more than 85% of all non-Hodgkin lymphomas; and their incidence has been rising steadily [1,2]. Aging of the population and increased life expectancy of the elderly is expected to result in B-cell malignancies, CLL in particular, becoming a progressively more common cause of morbidity and mortality in older individuals. Therapeutic MAbs against B-cell specific antigen CD20 are important players in the treatment of B-cell leukemia and lymphoma [2]. Mabs exert anti-tumor activity by harnessing the body’s own natural immune response, especially antibody-dependent cellular cytotoxicity (ADCC), complement-dependent cytotoxicity (CDC) requiring the activation of the classical complement pathway, and/or inducing apoptosis [3]. The widespread expression of CD20 on B-cells has resulted in the development of numerous antibodies. The first was rituximab, a human/murine chimeric Mab against CD20 [2,4,5]. Targeting CD20 in several hematologic malignancies proved to be an effective therapeutic approach that, eventually, led to the development of several more potent anti-CD20 antibodies with additional built-in mechanisms of action. Namely, ofatumumab is a next generation fully human anti-CD20 MAb that induces more potent CDC than rituximab [6,7,8]. Obinutuzumab is a next generation of type II glyco-engineered humanized anti-CD20 monoclonal antibodies, characterized by increased ADCC and efficient induction of direct non-apoptotic cell death [9].

Although the efficacy of MAbs is well established, many patients do not respond to the first treatment and others experience relapse after initial response to the therapy [10,11]. Rituximab and ofatumumab-mediated CDC is challenged by complement depletion, while patients with polymorphism of the Fcγ IIIa receptor on cytotoxic cells are less sensitive to antibody-dependent cellular cytotoxicity. In addition, overexpression of anti-apoptotic Bcl-2 proteins such as BCL-XL leads to resistance to rituximab-induced apoptosis [12,13,14].

Prostaglandin EP4 receptor is a negative feedback regulator of B-cell proliferation in response to B-cell receptor (BCR)-signaling. Our previous studies have shown that the EP4 receptor agonist 1-hydroxy prostaglandin E1 (PgE1-OH) induced selective cytotoxicity toward malignant B-cells and inhibited the anti-apoptotic NF-κB-dependent signaling pathways in B-cell lymphoma, resulting in a decrease in anti-apoptotic protein BCL-XL and an increased caspase-mediated apoptosis of malignant B-cells [15,16,17,18]. Similarly, the EP4 receptor agonist L-902688 induced selective cytotoxicity toward B-cell leukemia and lymphoma cells, was shown to inhibit the NF-κB pathway, cell proliferation, and induced apoptosis of CLL cells [19]. Moreover, in combination with ibrutinib, idelalisib or venetoclax, L-902688 induced synergistic cytotoxic activity against patient-derived CLL cells [19].

B-cell malignancies remain largely incurable; a significant proportion of patients are non-responsive or relapse after initial therapy. In the search for innovative therapeutic approaches for B-cell leukemia and lymphoma, we evaluated the potential synergism of prostaglandin EP4 receptor agonists PgE1-OH and L-902688 with therapeutic MAbs. Herein, we report enhanced cytotoxicity of anti-CD20 MAbs rituximab, ofatumumab and obinutuzumab when applied together with PgE1-OH and L-902688, compared to cytotoxicity of each agent alone. To evaluate molecular mechanisms, Ramos cells were used as a well-established model. To mimic resistant CLL, fludarabine-resistant cell line MEC-1 was utilized. The enhanced cytotoxic effects of EP4 receptor agonist PgE1-OH combined with anti-CD20 MAbs were also confirmed on primary cells obtained from patients diagnosed with CLL. This study reveals a substantial value of combining PgE1-OH and L-902688 with anti-CD20 MAbs, which may contribute to the development of novel therapies for patients with B-cell malignancies.

## 2. Results

### 2.1. Diverse Induction of Cell Death by Anti-CD20 MAbs

Rituximab, ofatumumab, and obinutuzumab are therapeutic anti-CD20 antibodies exploited for the treatment in a variety of B-cell malignancies, including CLL. Unresponsiveness of patients to the antibodies includes the exhaustible complement system, as CDC is one of the major mechanisms mediating MAbs tumoricidal activity in vivo. To address the issue of resistance, the well-established B-cell lymphoma cell line Ramos, as well as the p53-deficient CLL cell line MEC-1, were utilized.

To investigate the mode of cell death induced by therapeutic MAbs, growth media was supplemented with human serum as a source of complement components. Ramos and MEC-1 cell were, thus, incubated in the presence of up to 20% active human serum and therapeutic concentration, i.e., 10 µg/mL. The three tested MAbs show diverse CDC in vitro, which is furthermore cell dependent, since the responses varied significantly between Ramos and MEC-1 cells.

**Figure 1:**
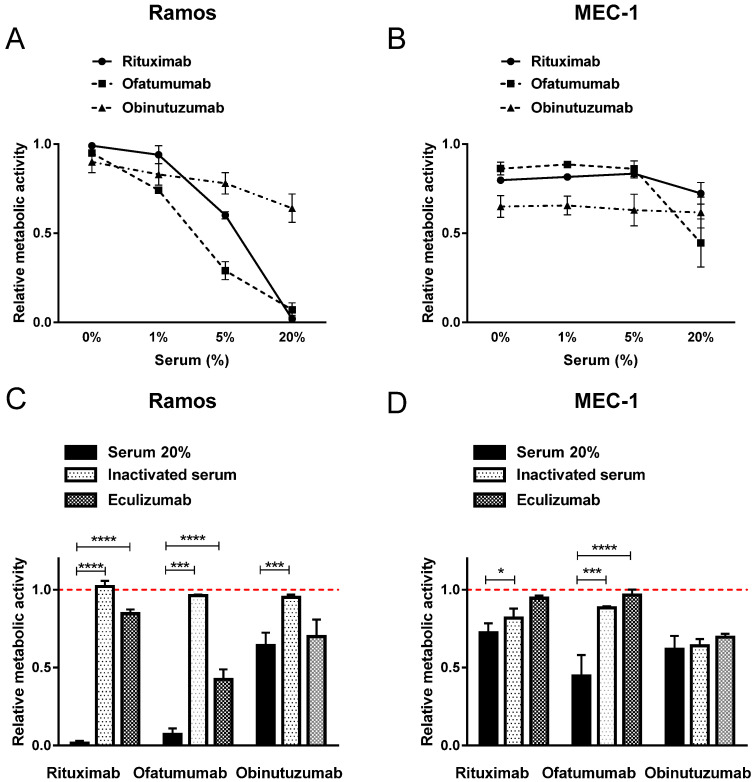
Anti-CD20 MAbs rituximab and ofatumumab induce CDC while obinutuzumab is less effective. The effect of active human serum on MAbs induced CDC was evaluated in Ramos (**A**) and MEC-1 (**B**) cells. The cells were incubated with a therapeutic concentration of 10 µg/mL of rituximab, ofatumumab or obinutuzumab in the presence of 1%, 5% or 20% active human serum and their metabolic activities were determined after 24 h. MAbs-induced CDC was assessed in Ramos (**C**) and MEC-1 (**D**) cells. The cells were incubated in the presence of 20% active human serum, heat deactivated human serum or 20% active human serum and eculizumab. Metabolic activity was determined after 24 h. Red dotted line represents the relative metabolic activity of untreated control cells. Data are presented as ratios relative to untreated control cells (mean ± SD of three independent experiments performed in triplicate). ANOVA, with post hoc analysis using Tukey’s multiple comparison, * *p* ˂ 0.05, *** *p* ˂ 0.001, **** *p* ˂ 0.0001.

In Ramos cells, all three MAbs induced serum-concentration dependent cytotoxicity when compared to untreated control cells. Rituximab and ofatumumab completely diminished viable cells in the presence of 20% active human serum. Obinutuzumab, however, was less potent in the presence of 20% human serum, as cell viability fell to 64% (Figure 1A). A striking difference in efficiency of MAbs was, however, observed in MEC-1 cells. The viability decreased to 72% in rituximab-, to 45% in ofatumumab- and to 61% in Obinutuzumab-treated MEC-1 cells in the presence of 20% human serum (Figure 1B). All three MAbs were shown to induce direct cell death in absence of serum in MEC-1 cell line, as evident in Figure 1B. The viability decreased to 80% in rituximab-, to 86% in ofatumumab- and to 65% in Obinutuzumab-treated MEC-1 cells (Figure 1B). These effects were less pronounced in Ramos cells and were in the range of 85% (Figure 1A).

Next, we assessed the viability of cells incubated with MAbs in conditions mimicking deprived complement components in vitro, which are often the reason for ineffectiveness of MAbs in vivo. Hence, heat-inactivated human serum containing denatured complement components as well as MAb eculizumab, which inhibits terminal complement activation, were utilized. Our findings confirmed that CDC is a key cell death mechanism of type I MAbs rituximab and ofatumumab (Figure 1C,D). Namely, only minor effects on cell viability (maximal 18% reduction) were observed when MEC-1 cells were cultured with rituximab and ofatumumab in the presence of heat-inactivated human serum, which is comparable to the effects of MAbs in the absence of human serum (Figure 1D). Similar effects with a trend for stronger cytotoxicity of both type I anti-CD20 MAbs were detected in Ramos cells (Figure 1C). Likewise, inhibition of terminal complement activation by eculizumab in the presence of 20% active human serum prevented CDC that is induced by rituximab and by ofatumumab in MEC-1 and Ramos cells (Figure 1C,D). On the contrary, strong cytotoxic effects were detected when MEC-1 cells were incubated with obinutuzumab in the absence of active human serum (Figure 1B). The latter was comparable to the effects of obinutuzumab incubated in the presence of 20% active human serum, in the presence of inactivated human serum, as well as to those of eculizumab, indicating direct cell death occurred (Figure 1D).

### 2.2. EP4 Receptor Agonists PgE1-OH and L-902688 Induce Time- and Concentration-Dependent Cytotoxic Effects via EP4 Receptor Activation

The EP4 receptor was recognized as a prospective target in B-cell leukemia and lymphoma. Hence, we evaluated the anticancer potential of PgE1-OH and L-902688 on Ramos and MEC-1 cells. We first determined the IC50 of PgE1-OH and L-902688 on MEC-1 cells. A time- and dose-dependent decrease in metabolic activity was determined for both compounds. Hence, IC50 values for PgE1-OH fell to 36.3 µM and 21.5 µM after 24 h and 48 h, respectively, whereas the IC50 values for L-902688 were 7.3 µM and 5.2 µM after 24 h and 48 h, respectively (Table 1). To confirm that the reduction in metabolic activity was due to cell death, cells were stained with PI. IC50 values obtained by this method were comparable to IC50 values determined by MTS assay. Next, the cytotoxic activities for both compounds were further determined on Ramos cells (Table 1); both compounds demonstrated time- and dose-dependent cytotoxicity also on Ramos cells, as expected and previously published [18,19].

**Figure 2:**
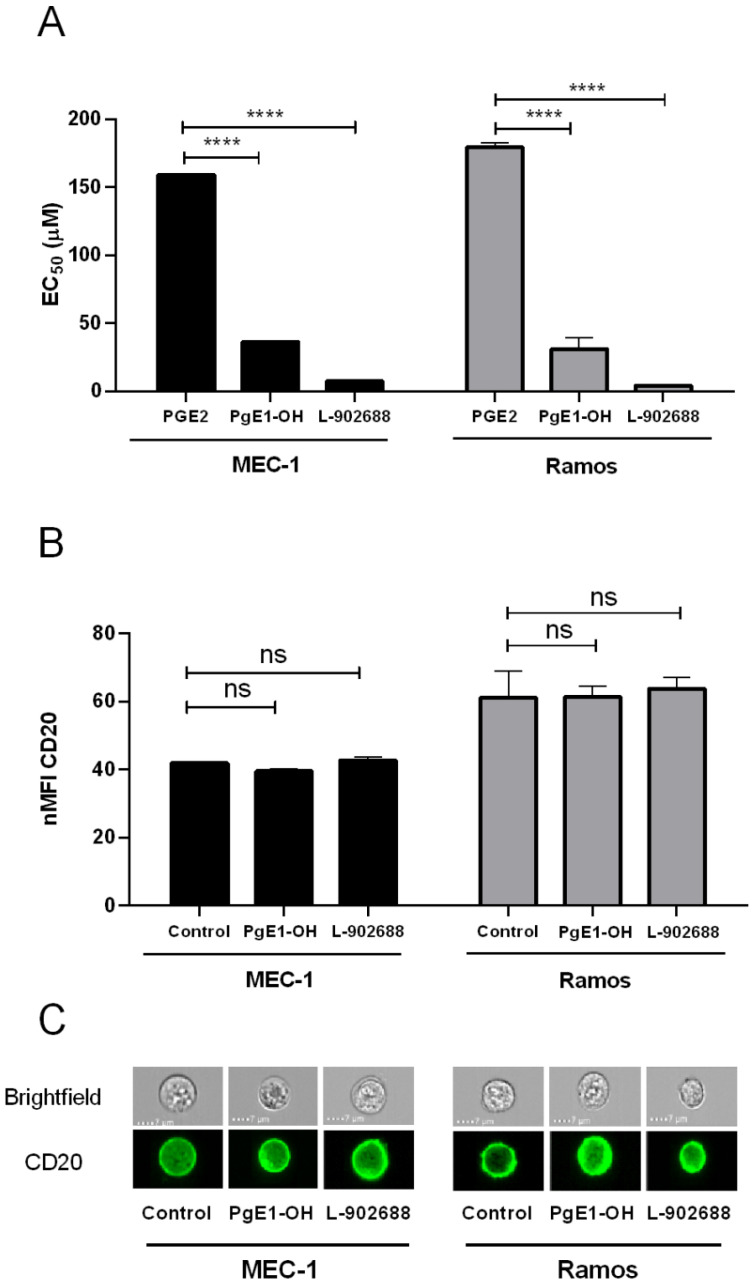
The cytotoxic effects of PgE1-OH and L-902688 are transduced via EP4 receptor. (**A**) MEC-1 and Ramos cells were exposed to increasing concentrations of PGE2, PgE1-OH, L-902688 (i.e., 1, 5, 10, 25, 50, 100, 150 μM). After 24 h, the metabolic activity of cells was determined and the IC50 values were calculated. Data are presented as ratios relative to untreated control cells (mean ± SD of three independent experiments performed in triplicate). Student’s *t*-test, ****, *p* < 0.0001, *ns, not significant*. (**B**) CD20 expression is preserved after EP4 receptor triggering. CD20 expression was determined in MEC-1 and Ramos cells. Cells were treated with non-cytotoxic concentrations of PgE1-OH (5 µM) and L-902688 (1 or 2.5 µM) for 24 h or left untreated (control cells). Median fluorescence intensity (MFI) of CD20 antigen was evaluated by flow cytometry (mean ± SD of three independent experiments). Student’s *t*-test, ns denotes *p* ≥ 0.05. (**C**) The expression of CD20 was determined by imaging flow cytometer and the images of representative cells are presented. Scale bar indicates 7 µm.

To further confirm the role of the EP4 receptor in malignant B-cells, we compared the effects of selective EP4 receptor agonists PgE1-OH and L-902688 to the EP4 receptor’s endogenous ligand prostaglandin E2 (PGE2), which binds to all 4 subtypes of prostaglandin E2 receptors. This indicated that the cytotoxic effects of PgE1-OH and L-902688 are due to selective agonism for the EP4 receptor, as PgE1-OH was 5-fold more cytotoxic and L-902688 was more than 22-fold more cytotoxic than the nonselective endogenous ligand PGE2 (Figure 2A). Thus, this supports our previous findings that the cytotoxic effects of PgE1-OH and L-902688 are transduced exclusively via the prostaglandin EP4 receptor [19].

Since CDC is a key mechanism leading to anti-CD20 MAbs’ tumoricidal activity, binding of therapeutic MAbs to CD20 on tumor cells is a critical factor for effective treatment. We next investigated whether EP4 receptor activation alters CD20 expression (Figure 2B,C). The data revealed that there was no significant difference between CD20 expression, as determined by flow cytometry on cells treated with PgE1-OH or L-902688 compared to untreated control cells. This indicates that activation of the EP4 receptor refrains from altering CD20 expression, thereby providing evidence for its beneficial, druggable profile in combination with anti-CD20 MAbs.

**Figure 3:**
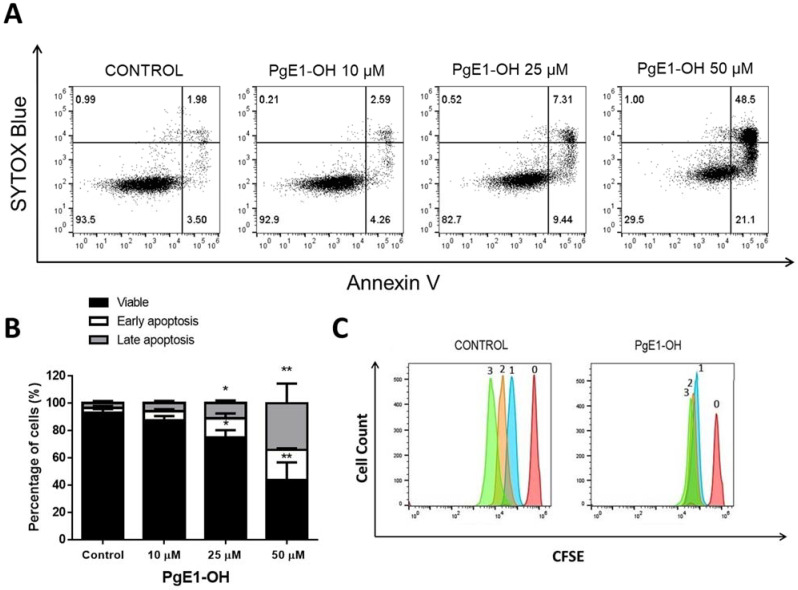
PgE1-OH induces apoptosis in CLL cells MEC-1. (**A**) Dose-dependent increases in the percentage of cells undergoing early and late apoptosis as compared to the untreated control. Cells were stained with Annexin V and SytoxBlue. Cells in the lower left quadrant are Annexin V-negative/SytoxBlue-negative (viable), cells in the lower right quadrant are Annexin V-positive/SytoxBlue-negative (pro-apoptotic), whereas cells in the upper right quadrant are Annexin V-positive/SytoxBlue-positive (late apoptotic). (**B**) Percentage of cells undergoing early apoptosis induced by PgE1-OH (mean ± SEM of two independent experiments). One-way ANOVA, with post hoc analysis using Tukey’s multiple comparison, *, *p* ˂ 0.05, ** *p* ˂ 0.01. (**C**) Anti-proliferative activity of PgE1-OH on MEC-1 cells. MEC-1 cells were stained with CFSE dye and incubated with 10 μM PgE1-OH or the vehicle treated control (0.1% DMSO) for 0, 24, 48 or 72 h. The proliferation of PgE1-OH-treated and untreated (0.1% DMSO) cells was analyzed by flow cytometry. Peaks 0, 1, 2, 3 represent fluorescence of CFSE stained cells after incubation periods of 0, 24, 48 or 72 h, respectively.

Our previous research revealed that EP4 receptor agonist PgE1-OH is a potent inducer of apoptosis and retains anti-proliferative activity in Burkitt lymphoma cells Ramos, and that L-902688 inhibits proliferation and induces a caspase-mediated apoptosis in the MEC-1 cell line [18,19]. Herein, we show that PgE1-OH triggers cell death by apoptosis. Hence, MEC-1 cells were stained with Annexin V, which binds to membrane exposed phosphatidylserine, a marker of apoptotic cell death, as well as to Sytox Blue, a nucleic acid stain that penetrates only the membranes of dead cells. PgE1-OH increased Annexin V single positive, i.e., pro-apoptotic cells and Annexin V/Sytox Blue double positive, i.e., dead cells at the expense of Annexin V/Sytox Blue double negative, i.e., live cells, when compared to the untreated control (Figure 3A). The increases in the percentage of early apoptotic cells treated with 10 μM, 25 μM or 50 μM PgE1-OH were 12.4%, 25.0% and 56.4%, respectively, compared to 7.4% in the untreated control (Figure 3B). PgE1-OH triggered a significant dose-dependent increase in the percentage of pro-apoptotic cells (*p* ˂ 0.05), confirming apoptosis as a mode of programmed cell death in MEC-1 cells. Effects are representative also for L-902688, which is in agrement with previously published results [19].

Since EP4 receptor activation triggered cell death, we next investigated whether lower concentrations of PgE1-OH cause inhibition of cell proliferation. MEC-1 cells were labelled with the CFSE probe and the transmission of the incorporated probe from parent to daughter generation was monitored. CFSE-labelled cells were treated with subIC50 10 μM concentration of PgE1-OH for 24 h, 48 h or 72 h. PgE1-OH completely inhibited the proliferative activity of MEC-1 cells (Figure 3C). PgE1-OH-treated cells were subjected to one cell division, whereas untreated cells divided two times during 72 h. Overlapping of the peaks after 24 h, 48 h and 72 h incubation led us to the conclusion that PgE1-OH exhibits strong anti-proliferative activity even at subIC50 concentrations. This data further confirms the potential of EP4 receptor agonist PgE1-OH in the treatment of B malignancies, including CLL.

### 2.3. EP4 Receptor Agonists PgE1-OH and L-902688 Enhanced the Cytotoxic Potential of Anti-CD20 MAbs Rituximab and Ofatumumab in Burkitt Lymphoma Cells Ramos

As evidenced herein, the MAbs-induced cell death is mainly dependent on the complement availability (Figure 1). This is, however, often compromised in B-cell leukemia and lymphoma patients due to exhausted complement components. It is, therefore, of prime importance to find novel paths to amplify MAbs effectiveness. We have, therefore, postulated that EP4 receptor agonists may augment MAbs activity.

**Figure 4:**
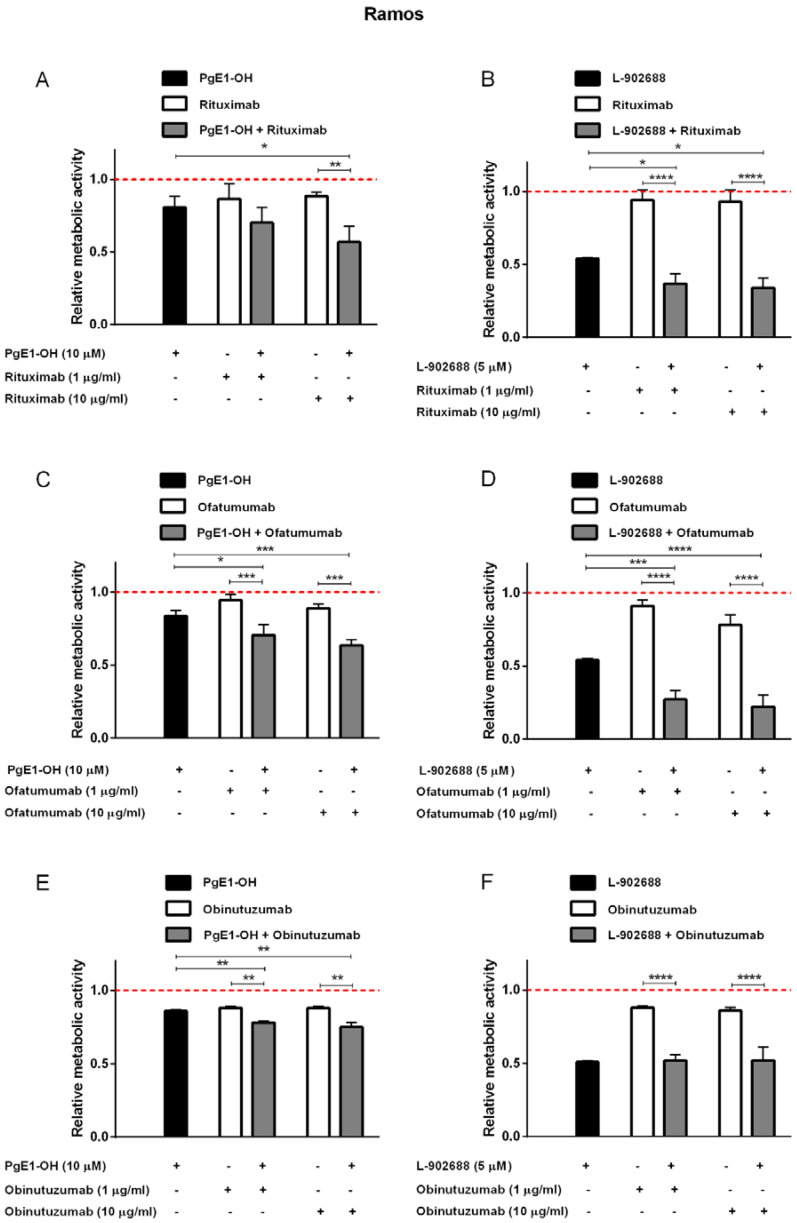
EP4 receptor agonists enhance the cytotoxicity of anti-CD20 MAbs against Burkitt lymphoma cell line Ramos. Cells were incubated with 10 μM PgE1-OH (**A**,**C**,**E**), 5 μM L-902688 (**B**,**D**,**F**) and 1 μg/mL or 10 μg/mL rituximab (**A**,**B**), ofatumumab (**C**,**D**) or obinutuzumab (**E**,**F**) in the presence of 1% human serum. Metabolic activity was determined after 48 h. Red dotted line represents the relative metabolic activity of untreated control cells. Data are presented as ratios relative to untreated control cells (mean ± SD of three independent experiments performed in triplicate). ANOVA, with post hoc analysis using Tukey’s multiple comparison, *, *p* ˂ 0.05, ** *p* ˂ 0.01, *** *p* ˂ 0.001, **** *p* ˂ 0.0001.

First, the effects of PgE1-OH and L-902688 in combination with MAbs targeting CD20 were investigated in Burkitt lymphoma cells Ramos under conditions mirroring a deprived complement system, i.e., in the presence of 1% human serum as a source of complement components. Ramos cells were treated with different concentrations of anti-CD20 MAbs rituximab, ofatumumab and obinutuzumab in therapeutic (10 μg/mL) and 10-fold lower (1 μg/mL) concentration individually in the absence and presence of 10 µ PgE1-OH or 5 µM L-902688. Treatment of Ramos cells with PgE1-OH enhanced the cytotoxicity of rituximab. As shown in Figure 4A, cell viability was reduced by 19% when Ramos cells were treated with 10 µM PgE1-OH. Rituximab, in a clinically relevant concentration (10 μg/mL), decreased cell viability by 11%, while the combination of both agents together further reduced cell viability by 43%. Interestingly, a subtherapeutic concentration of rituximab (1 μg/mL) achieved similar effects. To prove synergistic activity, the data were further assessed by the CI method of Chou and Talalay using the CompuSyn software, where CI ˂ 1 implies synergism, CI = 1 indicates additive effects and CI ˃ 1 implies antagonism. The calculated CIs were <0.6 for both combinations of PgE1-OH with rituximab, proving synergism (Table 2). Similarly, the viability was assessed after treatment of Ramos cells with L-902688 (5 µM,) rituximab (1 or 10 μg/mL) and their combinations (Figure 4B). L-902688 reduced the viability of cells by 46%, rituximab in clinically relevant concentration (10 μg/mL) by 7% and their combination by 66%. It is of importance to notice that 10-fold lower rituximab concentration (1 μg/mL) induced similar effects. Synergistic effects were observed also for PgE1-OH and ofatumumab (Figure 4C). The presence of a clinically relevant concentration of ofatumumab (10 μg/mL) led to a reduction in cell viability by 17% and the presence of 10 µM PgE1-OH by 16%. The addition of 10 µM PgE1-OH to the ofatumumab potentiated reduction in cytotoxic activities by 39%. The synergism between ofatumumab and PgE1-OH was again confirmed by CompuSyn calculation, where CI was <0.6 (Table 2). Moreover, L-902688, proved more potent than PgE1-OH and also enhanced the cytotoxicity of ofatumumab (Figure 4D). Ofatumumab in a clinically relevant concentration (10 μg/mL) reduced cell viability by 17%, the presence of 5 µM L-902688 by 46% and their combination by 78%. The additive effects of both agents together were confirmed since the calculated CI were ±1.0 (Table 2).

Taken together, while both rituximab and ofatumumab significantly enhanced the pharmacological effects of both EP4 receptor agonists, the synergistic effects were not detected with obinutuzumab on Ramos cells (Figure 4E,F).

### 2.4. EP4 Receptor Agonists PgE1-OH and L-902688 Enhanced the Cytotoxic Potential of Anti-CD20 MAbs in CLL Cells MEC-1

**Figure 5:**
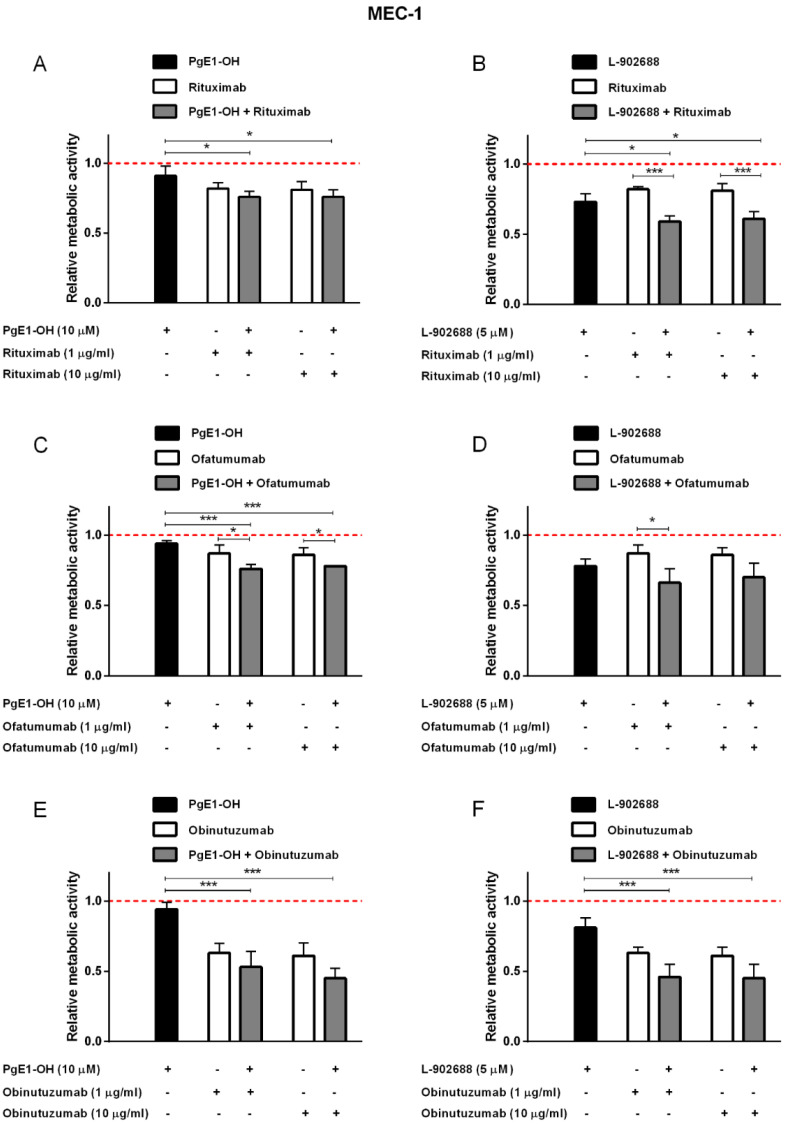
EP4 receptor agonists enhance cytotoxicity of anti-CD20 MAbs against the CLL cell line MEC-1. Cells were incubated with 10 μM PgE1-OH (**A**,**C**,**E**), 5 μM L-902688 (**B**,**D**,**F**) and 1 or 10 μg/mL rituximab (**A**,**B**), ofatumumab (**C**,**D**) or obinutuzumab (**E**,**F**) in the presence of one percent human serum. Metabolic activity was determined after 48 h. Red dotted line represents the relative metabolic activity of untreated control cells. Data are presented as ratios relative to untreated control cells (mean ± SD of three independent experiments performed in triplicate). ANOVA, with post hoc analysis using Tukey´s multiple comparison, *, *p* ˂ 0.05, *** *p* ˂ 0.001.

To delineate cell specific effects, we next investigated whether simultaneous activation of the EP4 receptor by PgE1-OH or L-902688 and targeting of CD20 antigen by MAb leads to an enhanced cytotoxic effect in p53-deficient CLL cells MEC-1. To mimic the conditions of depleted complement components commonly occurring in CLL in vivo, one percent human serum was added to the MEC-1 cell culture. The results revealed that PgE1-OH and L-902688 treatments of MEC-1 cells synergistically enhanced rituximab´s cytotoxicity (Figure 5A,B, Table 2). In agreement with results obtained on Ramos cells, the subtherapeutic concentration of rituximab induced similar synergistic effects to 10-fold higher than the therapeutic concentration; however, the effects of combined treatment were less pronounced on MEC-1 cells compared to Ramos cells. The synergistic effects of PgE1-OH and L-902688 with anti-CD20 monoclonal antibody ofatumumab were also detected, as shown in Figure 5C,D.

The synergistic effects on MEC-1 cells were also proven when PgE1-OH was combined with obinutuzumab, a novel anti-CD20 MAb designed for the treatment of CLL. Obinutuzumab (10 µg/mL) decreased cell viability by 39%, the presence of 10 µM PgE1-OH by 6% and their combination by 55% (Figure 5E). These results were even more enhanced when MEC-1 cells were incubated by increased concentration of PgE1-OH (25 µM), leading to a 62% reduction in cell viability after treatment with a therapeutic concentration of obinutuzumab (data not shown). Very strong synergistic action of both agents was determined in therapeutic as well as in a 10-fold lower concentration of obinutuzumab. Respective synergy scores were high (CI < 0.1) (Table 2). Similarly, cell viability was assessed after treatment of MEC-1 cells with L-902688 (5 µM), obinutuzumab (1 or 10 mg/mL) and their combinations (Figure 4F). L-902688 reduced the viability of cells by 19%, the therapeutic concentration of obinutuzumab (10 μg/mL) by 39% and their combination by 55%. The synergism between obinutuzumab and L-902688 was confirmed by CompuSyn calculation, since CI values were <0.4. Hence, combined treatment with EP4 receptor agonists and anti-CD20 MAbs could greatly improve the outcomes in B-cell CLL.

### 2.5. Anti-CD20 MAbs and PgE1-OH Act Synergistically Cytotoxic in Primary CLL Cells

Despite the transformed cell lines representing a valuable in vitro model for studying the efficacy of novel therapeutic options, their ability to simulate complex pathophysiological conditions and heterogeneity representative of B-cell malignancies, such as CLL, is limited. Thus, the effects of EP4 receptor agonists in a combination with MAbs targeting CD20 were further investigated on primary CLL cells obtained from patients diagnosed with CLL in an ex vivo assay.

**Figure 6:**
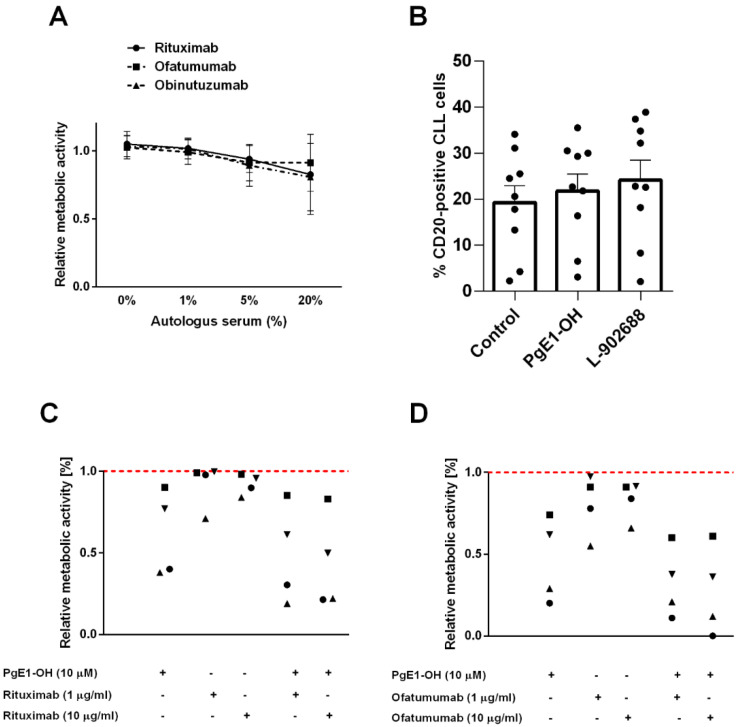
EP4 receptor agonist-induced cytotoxicity in primary CLL cells is augmented by anti-CD 20 MAbs rituximab and ofatumumab. (**A**) The degree of MAbs-induced CDC was evaluated in primary CLL cells (*n* = 7). The impact of concentration of autologous human serum on MAbs-induced CDC was determined by cultivating the cells at therapeutic concentrations of 10 μg/mL of rituximab, ofatumumab and obinutuzumab in the presence of 1%, 5% and 20% autologous serum. Metabolic activity was determined after 48 h. Data are presented as ratios relative to untreated control cells (mean ± SD, from seven samples of unrelated CLL donors, performed in triplicate). (**B**) CD20 expression was determined in primary CLL cells (*n* = 9). Cells were treated with non-cytotoxic concentrations of (10 µM) and L-902688 (2.5 µM) for 24 h or left untreated (control cells). Percentage of CD20 antigen positive cells was evaluated by flow cytometry (mean ± SEM, from nine CLL samples). Synergistic effects of PgE1-OH and (**C**) rituximab, (**D**) ofatumumab in primary CLL cells. CLL cells were incubated with PgE1-OH (10 μM) and MAbs (1 μg/mL, 10 μg/mL) in the presence of one percent autologous human serum. Metabolic activity was determined after 48 h. The experiments were performed in triplicate. Red dotted line represents the relative metabolic activity of untreated control cells. Data are presented as ratios relative to untreated control cells. Each sign denotes a sample of a representative CLL donor (*n* = 4).

To estimate the extent of CDC occurring ex vivo, primary CLL cells were exposed to therapeutic concentrations of MAbs in the presence of 1%, 5% and 20% autologous serum (*n* = 7). The increasing concentrations of autologous serum resulted in only moderate induction of CDC, i.e., with viability exceeding 80% in the presence of 20% autologous serum ex vivo (Figure 6A).

Since binding of therapeutic MAbs to CD20 on tumor cells is a critical factor for effective treatment, we determined CD20 expression in primary CLL cells after incubation with non-cytotoxic concentrations of PgE1-OH (10 µM) and L-902688 (2.5 µM) (Figure 6B). The data revealed that PgE1-OH and L-902688 did not significantly affect the expression of CD20 in several primary CLL samples. Hence, these results alleviate concerns about the potential usefulness of combining EP4 receptor agonists with anti-CD20 MAbs in therapy of B-cell leukemia and lymphoma.

As expected, patient-derived CLL cells possess large inter-individual variablity in responses to both MAbs and EP4 receptor agonists. The cells obtained from four patients diagnosed with CLL were incubated with 10 μM PgE1-OH, rituximab (1 μg/mL, 10 μg/mL) or their combinations (Figure 6C). While inter-individual differences in response to both agents were observed in all four CLL samples tested (Supplementary information, Figure S1), the same trend for synergy was detected. Treatment with rituximab alone had on average negligible effects on cell viability with only an 8% reduction observed. PgE1-OH reduced cell viability on average by 39%, while both agents combined reduced the percentage of viable cells by 55%. Next, anti-CD20 MAb ofatumumab was assessed in combination with PgE1-OH (Figure 6D). The same trend for synergy was detected in all four primary CLL samples, while inter-individual differences in response to both agents were observed (Supplementary information, Figure S2). Ofatumumab in a clinically relevant concentration (10 μg/mL) decreased cell viability, on average, by 17%, while 10 µM PgE1-OH decreased it by 54% and both agents together by 73%, clearly indicating synergistic action. Taken collectively, this data shows that combining PgE1-OH with rituximab as well as ofatumumab could provide a beneficial therapeutic effect in CLL patients. Under investigated experimental conditions, obinutuzumab did not show synergistic effects. Similar synergistic effects with targeted therapy were observed also for L-902688 [19].

## 3. Discussion

Anti-CD20 MAbs provide a significant therapeutic benefit for patients with B-cell disorders. Since the responses to the therapy vary and relapses are common, there is a need for novel therapeutic approaches to bypass innate and acquired resistance. We have previously shown that EP4 receptor agonists are potent inducers of apoptosis in malignant B-cells [18,19].

The aim of this study was to evaluate the potential synergistic effects of therapeutic MAbs and EP4 receptor agonists PgE1-OH and L-902688 in B-cell leukemia and lymphoma. Herein, we describe, for the first time, the synergistic effects of the selective EP4 receptor agonists PgE1-OH and L-902688 and anti-CD20 MAbs, leading to enhanced cytotoxicity in malignant B-cells in vitro and ex vivo.

PGE2 is a potent endogenous molecule that binds to four different G-protein coupled prostaglandin receptors, EP1 to 4, each with distinct tissue localizations and different signaling pathways. The EP4 receptor represents an important drug target with novel EP4 receptor agonists and antagonists being evaluated in early phase clinical trials [17,20]. In line, PgE1-OH was previously assessed in clinical trial for the treatment of asthma patients and was well tolerated, confirming its value as a potential safe therapeutic compound [21,22]. L-902688 is a highly potent EP4 receptor agonist, which demonstrated several favorable pharmacokinetic parameters in vivo and has been evaluated in preclinical studies in ischemic stroke, pulmonary hypertension and asthma [23,24,25,26,27,28,29]. Our previous studies identified that PgE1-OH and L-902688 induced selective cytoxicity toward B-cell leukemia and lymphoma cells at low micromolar concentrations. EP4 receptor triggering decreased NF-κB activity, subsequently diminishing levels of anti-apoptotic protein BCL-XL [15,16,17,18,19,30].

In this study we provide additional evidence that PgE1-OH- and L-902688-induced cytotoxicity is transduced via EP4 receptor in malignant B-cells. Namely, the endogenous ligand PGE2, which binds to four prostaglandin EP receptors, exhibited no cytotoxic effect at the lower concentrations tested in MEC-1 and Ramos cells, while the same concentrations of EP4 receptor agonists, PgE1-OH and L-902688, induced notable cytotoxicity. IC50 values determined for PGE2 were approximately 5-fold higher and more than 22-fold higher than those obtained for PgE1-OH and L-902688, respectively. In our previous study EP4 receptor antagonist CJ-042794 suppresed the L-902688-mediated inhibitory effects on CLL cell viability [19]. The results obtained in this study, thus, support our previous findings and indicate EP4 receptor triggering as the exclusive transducer of cytotoxic activity of PgE1-OH and L-902688.

Herein, we demonstrate that the EP4 receptor agonist PgE1-OH also induced cell death in primary B-cells obtained from patients diagnosed with CLL, as well as CLL cell line MEC-1 via apoptosis. Moreover, we demonstrated that PgE1-OH completely inhibited the proliferative activity of MEC-1 cells. This further endorses the potential of the EP4 receptor agonist in the B-cell malignancies, including CLL, since it elicits both cytotoxic and anti-proliferative activities in MEC-1 cells.

MAbs, alone or in combination with chemotherapy, have revolutionized the treatment of non-Hodgkin´s lymphoma and CLL. In spite of that, most B-cell malignancies remain incurable and a significant proportion of the patients still relapse. In follicular lymphoma, only half of these patients respond to initial treatment with single-agent rituximab [10] and the majority of responders eventually become refractory [11,14]. The expression of complement-defense molecules and complement exhaustion after anti-CD20 MAb infusions are associated with its resistance [3,31,32]. Analyses of serum samples obtained from 63 CLL patients revealed that 38% were deficient in one or more complement components, correlating with reduced CDC responses [3]. This notion was confirmed in our study, since the presence of 20% autologous serum resulted in only minor induction of CDC by all three MAbs tested in primary CLL cells. This corresponds with the fact that CLL patients are often deprived of a functional complement system. In line, replacement of the consumed components restores the activity of rituximab in ex vivo assays, which indicates CDC as one of the key effector mechanisms [31,32,33]. Moreover, ofatumumab induced stronger CDC compared to rituximab, which is in line with the fact that ofatumumab has novel epitope binding side with improved binding to C1q, resulting in enhanced CDC [33].

Moreover, we demonstrated that all three MAbs exhibited no or notably attenuated effects on the viability of Burkitt lymphoma cells Ramos upon deprivation of complement components as compared to incubation with functional human serum. These data further corroborate the facts that patients with exhausted complement system components or higher expression of complement inhibitory proteins have lower response rates to anti-CD20 MAbs, particularly to rituximab and ofatumumab [3,31,34].

Using MEC-1 and Ramos cells, we confirmed that the therapeutic MAbs rituximab and ofatumumab induced CDC in the presence of functional human serum, while obinutuzumab is a strong inducer of direct cell death in vitro. To explain the observed differences in MAbs activity, MEC-1 and Ramos cells were probed for expression of CD20. Higher expression of CD20 molecules on Ramos than of MEC-1 cells resulted in augmented anti-CD20 MAbs-mediated CDC. In line with this, CD20 expression levels are relatively low in CLL compared with those in B-cell lymphomas and were shown to correlate linearly with the lytic response of rituximab and ofatumumab [3,14]. This was confirmed in our study, in which CD20 expression levels were higher on Burkitt lymphoma cells Ramos than on CLL cells MEC-1 and on primary CLL cells.

Since CDC is a key mechanism responsible for MAbs´ tumoricidal activity, their binding to CD20 on tumor cells is a critical factor for effective treatment. Recently, gemcitabine-induced CD20 upregulation was reported to lead to enhanced rituximab-mediated CDC [35]. On the contrary, BCR pathway inhibitor ibrutinib decreased CD20 expression in malignant B-cells, leading to decreased rituximab and ofatumumab-induced CDC [36]. To address the impact of the EP4 receptor triggering on CD20 modulation, we determined CD20 expression levels on Ramos, MEC-1 and primary CLL cells upon treatment with PgE1-OH or L-902688. Compared to untreated control cells, flow cytometry analysis revealed no significant differences in CD20 expression when all cells were incubated with PgE1-OH. Similarly, treatment of Ramos and MEC-1 cells with L-902688 resulted in no significant differences in CD20 expression, while the treatment of primary CLL cells with L-902688 slightly increased the expression of CD20 in several primary CLL samples. This observation further endorsed the potential of the EP4 receptor triggering in combination with anti-CD20 MAbs. Along this line, the effects of PgE1-OH and L-902688, in combination with therapeutic MAbs targeting of CD20, were investigated in Burkitt lymphoma and CLL cells.

Indeed, the synergistic effects of MAbs with EP4 agonists were present in both MEC-1 and Ramos cells. Co-treatment of Ramos cells with PgE1-OH and L-902688, in combination with rituximab and ofatumumab, led to augmented cytotoxic effects. The synergistic effects may be the result of targeting important, yet different mechanisms in CLL cells, namely induction of apoptosis by EP4 receptor agonists as well as CDC by anti-CD20 MAbs rituximab and ofatumumab. Moreover, EP4 receptor-mediated BCL-XL down-regulation may chemo-sensitize the cells to anti-CD20 MAbs induced apoptosis [18,19]. Moreover, very strong synergism was detected when obinutuzumab was combined with PgE1-OH or L-902688 in MEC-1 cells. This is in accordance with the fact that obinutuzumab is glycoengineered type II MAb registered for the treatment of CLL, triggering antibody dependent cell cytotoxicity as well as direct non-apoptotic cell death [9,33,37]. In particular, the action of obinutuzumab in causing cell death lies in the induction of homotypic aggregation, a novel type of actin-dependent and lysosome-induced cell death, associated with actin rearrangement and lysosome cathepsin release. Due to the lack of characteristic markers of apoptosis, such as caspase-dependence or BCL2 expression, this type of cell death can bypass the mechanism of resistance to apoptosis [9]. The synergistic effects identified in our study could, therefore, be the result of two independent, yet complementary cell death mechanisms, one induced by EP4 receptor triggering and the other by binding of therapeutic antibody to CD20 antigen, simultaneously causing apoptotic and direct non-apoptotic cell death, respectively.

Consequently, we validated the potential synergistic effects of simultaneously targeting CD20 antigen and EP4 receptor ex vivo, using primary lymphocytes B isolated from patients diagnosed with CLL. This represents a valuable insight into the pathophysiological conditions and heterogeneity of CLL. The results revealed that the addition of therapeutic anti-CD20 MAbs synergistically augmented PgE1-OH-induced cytotoxicity to primary CLL cells. While inter-individual differences were observed in response to PgE1-OH, as well as to rituximab and ofatumumab, the trend for greater efficiency when the EP4 receptor agonist and anti-CD20 MAb were combined was persistent in all samples tested. Treatment of primary CLL cells with PgE1-OH and rituximab or ofatumumab enhanced cytotoxicity through synergistic interactions. Moreover, for the first time, the anti-leukemic potential of EP4 receptor agonist PgE1-OH was confirmed in primary CLL cells.

Taken together, PgE1-OH and L-902688 increased the ability of rituximab and of ofatumumab to induce cell death in Ramos and MEC-1 cells. Furthermore, the synergistic effects of the EP4 receptor agonist PgE1-OH and anti-CD20 MAbs were verified on primary CLL cells. Our results are in line with numerous clinical and pre-clinical studies, confirming beneficial anti-cancer effects of ant-CD20 MAbs in combination with therapeutic compounds [38]. As MAbs are often used in combination with targeted therapies, we next aim to investigate how the addition of EP4 receptor agonist complements these combinations. What is even more important and needs to be addressed in the future is whether EP4 receptor agonists can overcome resistance of CLL cells to targeted therapy.

Our findings are thus of significant importance, as combining agents with synergistic action often results in favorable therapeutic outcomes for the patient. Moreover, introduction of two or more compounds with synergistic action may reduce therapeutic doses, which may in turn lead to less side effects. In addition, such combined therapy can protect patients from drug resistance [19,38].

In conclusion, our in vitro/ex vivo study highlights the benefits of combining anti-leukemic action induced by triggering of EP4 receptor and targeting of CD20 antigen in B-cell leukemia and lymphoma.

## 4. Materials and Methods

### 4.1. Materials

PgE1-OH, L-902688 and PgE2 were obtained from Cayman Chemical, Ann Arbor, MI, USA. Rituximab (MabThera) was obtained from Roche, Basel, Switzerland, ofatumumab (Arzzera) from Novartis, Basel, Switzerland, obinutuzumab (Gazyva) from Genentech, South San Francisco, CA, USA and eculizumab (Soliris) from Alexion, Cheshire, CT, USA.

### 4.2. Cell Culture

Ramos cell line (ATCC No.: CRL-1596) was purchased from ATCC (American Type culture Collection, Manassas, VA, USA) and was maintain:ed in an RPMI 1640 medium (SigmaAldrich, St. Louis, MO, USA) supplemented with 10% heat-inactivated fetal bovine serum (Gibco, Grand Island, NY, USA), 2 mM L-glutamine, 100 U/mL penicillin, 100 µg/mL streptomycin and 50 mM 2-mercaptoethanol (all from SigmaAldrich, St. Louis, MO, USA).

CLL cell line MEC-1 (DSMZ No.: ACC 497) was obtained from DSMZ (Deutsche Sammlung von Mikroorganismen und Zellkulturen, Braunschweig, Germany). MEC-1 cells were cultured in an IMDM medium (Sigma-Aldrich, St. Louis, MO, USA) supplemented with 10% heat-inactivated fetal bovine serum (Gibco, Grand Island, NY, USA), 100 U/mL penicillin and 100 µg/mL streptomycin.

All experiments were performed in accordance with the Declaration of Helsinki and approved national ethical guidelines. After obtaining informed consent in accordance with the ethical approval of the Republic of Slovenia National Medical Ethics Committee (Nr. 93/12/10, approved: 27 December 2010 and Nr. 0120-136/2019/4, approved: 19 March 2019), 5 mL of peripheral blood was collected from patients diagnosed with CLL at the University Medical Centre, Ljubljana, Slovenia. CLL patients were diagnosed on the basis of peripheral blood clonal lymphocytes with a concentration > 5 × 10^9^/L and immunophenotyping. Lymphocytes B from peripheral blood samples from CLL patients were isolated using the RosetteSep Ficoll-Paque (StemCell Technologies, Vancouver, Canada) procedure. Isolated CLL cells were washed in phosphate buffer saline (PBS) and counted. Isolated CLL cells were cultured in RPMI 1640 medium (Sigma-Aldrich, St. Luis, MO, USA) supplemented with 10% heat-inactivated fetal bovine serum (Gibco, Grand Island, NY, USA), 2 mM L-glutamine, 100 U/mL penicillin, 100 µg/mL streptomycin and 50 mM 2-mercaptoethanol. All cells were cultured at 37 °C in a humidified atmosphere with 5% CO_2_.

### 4.3. Metabolic Activity Assay

The metabolic activities of the cell lines were assessed by measuring MTS reduction to a colored formazan dye in viable cells using the CellTiter 96^®^ Aqueous One Solution Cell Proliferation Assay (MTS reagents, Promega, Madison, WI, USA) according to the manufacturer’s instructions. Prior to each experiment, Ramos and MEC-1 cells were counted and diluted to a concentration of 3.0 × 10^5^ cells/mL. The cells were treated with appropriate amounts of the compounds of interest or the corresponding vehicle (vehicle control). Assays were performed in triplicate in 96-well plates. Absorbance was measured at 492 nm on an automated microplate reader Synergy™ HTX Multi-Mode Microplate Reader (BioTek Instruments, Inc., Winusky, VT, USA). Relative cell viability of treated cells was calculated by subtracting the absorbance values of the blank (medium) and by normalizing to the absorbance of vehicle-treated controls.

The metabolic activities of primary CLL cells were assessed by means of highly-sensitive resazurin-based assay using the PrestoBlue^®^ Cell Viability Reagent (ThermoFisher Scientific, Waltham, MA, USA) according to the manufacturer’s instructions. Prior to each experiment, CLL cells were counted and diluted to the concentration of 4.0 × 10^5^ cells/mL. The cells were treated with appropriate amounts of the compounds of interest or the corresponding vehicle (vehicle control). Assays were performed in triplicate in 96-well black plates. Fluorescence was measured at 590 nm on an automated microplate reader. Relative cell viability of treated cells was calculated by subtracting the fluorescence values of the blanks (medium), which contained no cells, and normalizing to the fluorescence of the vehicle-treated controls.

### 4.4. Viability Assessment Using Propidium Iodide (PI Exclusion Assay)

Propidium iodide (PI) staining followed by flow cytometric analysis was used to confirm the cytotoxicity of PgE1-OH and L-902688 on Ramos and MEC-1 cells, as observed using the metabolic activity assay MTS. Cells (3 × 10^5^ cells/mL) were treated with PgE1-OH, L-902688, 0.1% DMSO (control vehicle) or left untreated (control) and seeded in a duplicate, in a 96 well plate. After 48 h incubation, to each 100 μL of cell suspension 0.5 μL of PI (final concentration 5 μM) was added, followed by analysis using an Attune Nxt flow cytometer connected to Autosampler (Invitrogen, Waltham, MA, USA). Cells that stain positive for PI are considered dead. The result shows the viability which was normalized to that of vehicle-treated control cells, to produce relative viability (%).

### 4.5. CFSE Assay

The proliferation of MEC-1 cells was assessed using the CellTrace™ Cell Proliferation Kit (Invitrogen Molecular Probes, Carlsbad, CA, USA), following the manufacturer’s instructions. Cells were washed and resuspended in PBS at 1.0 × 10^6^ cell/mL and incubated with CFSE (final concentration 5 µM) for 15 min at 37 °C. Cells were washed and resuspended in fresh culture medium and incubated for 30 min at 37 °C for complete modification of the probe. After the final wash step, cells were resuspended in culture medium at 3 × 10^5^ cells/mL and treated with 10 µM PgE1-OH for 0, 24, 48, and 72 h. At the indicated time points, samples were examined using flow cytometry (Attune NxT, Invitrogen, Waltham, MA, USA).

### 4.6. Analysis of Apoptosis with Annexin V/Sytox Blue Staining

Apoptosis of MEC-1 cells treated with PgE1-OH was assessed using Annexin V, conjugated with R-phycoerythrin (R-PE) (R-PE Annexin V; Molecular Probes™, Eugene, OR, USA) and the DNA staining reagent Sytox Blue (Sytox™ Blue Dead Cell Stain; Molecular Probes™, Eugene, OR, USA). Prior to the experiment, cells were washed twice with PBS (5 min, 1000 rpm), and resuspended in culture medium at 3 × 10^5^ cells/mL concentration. Cells were incubated with PgE1-OH for 24 h (37 °C, 5% CO_2_). After 24 h, cells were washed twice with cold PBS (0 °C) and resuspended in Annexin-binding buffer (10 mM HEPES, 140 mM NaCl, 2,5 mM CaCl_2_, pH 7.4) to produce 1.0 × 10^6^ cells/mL. To each sample (100 μL), 2.5 μL R-PE Annexin V and 1 μL 10x diluted Sytox Blue (final concentration 1 μM) was added. Samples were incubated at room temperature for 15 min. After the incubation period, 200 μL of Annexin-binding buffer was added to each sample, followed by gently mixing and putting samples on ice. Apoptosis was measured by flow cytometry (Attune NxT, Invitrogen, Waltham, MA, USA). Cells that stained negative for both R-PE Annexin V and Sytox Blue were considered alive. Cells that stained positive only for R-PE Annexin V were considered in early apoptosis. Cells that stained positive for both R-PE Annexin V and Sytox Blue have a permeable membrane, and were, thus, designated as late apoptotic, i.e., dead. Cells staining positive only for Sytox Blue were undergoing primary necrosis.

### 4.7. CDC Assay

For analysis of CDC, cells were incubated with or without 10 µg/mL MAb for 48 h in the presence of 1%, 5% or 20% active human serum (Sigma Aldrich, St. Louis, MO, USA) as a source of complement. The proportion of lysed cells was assessed by MTS assay.

To inhibit CDC, cells were pre-treated for 1 h with 200 µg/mL eculizumab in the presence of 20% active human serum or incubated in 20% heat-inactivated human serum. Denaturation of complement components was performed by incubating human serum for 1 h at 56 °C. Cells were incubated with 10 µg/mL MAb for 48 h. The proportion of lysed cells was assessed by MTS assay.

### 4.8. Evaluation of CD20 Antigen Expression

CD20 mean fluorescence intensity (MFI) values were obtained from untreated (control cells) and PgE1-OH- and L-902688-treated Ramos, MEC-1 and primary CLL cells. For the purpose of CD20 analysis, treated cells were incubated with non-cytotoxic concentrations of PgE1-OH or L-902688 for 24 h. Briefly, cells were washed with 0.5 mL of PBS and resuspended in 100 µL of PBS. Cells were incubated in the dark for 15 min with 5 µL of purified mouse anti-human CD20 antibody conjugated with FITC (555622, BD Biosciences, San Jose, CA, USA). Cells were then centrifuged (200× *g*, 5 min) and resuspended in 200 µL of PBS. Cells were analyzed by flow cytometry using Attune Nxt (Invitrogen, Carlsbad, CA, USA) and FlowJo software (TreeStar, Ashland, OR, USA). Alternatively, the expression of CD20 was measured on Amnis^®^ ImageStream^®^X Mk II imaging flow cytometer (Luminex Corporation, Austin, TX, USA). A minimum of 10,000 events in focus was collected per sample.

### 4.9. Combination Index Calculation

Combination index (CI) values were calculated according to the Chou and Talalay mathematical model for drug interactions [39]. Dose-response curves, dose-effect analysis and CI for the combination treatment groups were generated using the equations reported by Chou and Talalay, using the CompuSyn software (freely available at: www.combosyn.com (accessed on 20 October 2020). CI ˂ 1 implies synergism; CI ± 1 indicates additive effects; and CI ˃ 1 implies antagonism.

### 4.10. Data Analysis

Statistical analysis was performed using the GraphPad Prism software (GraphPad Software, Inc., La Jolla, CA, USA). Data were analyzed by Student’s t-tests and one-way or two-way analysis of variance (ANOVA); differences between treatments were established via Tuckey´s post hoc multiple comparison test. Means were considered statistically significant for *p* < 0.05 and highly significant for *p* < 0.01 and *p* < 0.001.

## Figures and Tables

**Table 1:** EP4 receptor agonists PgE1-OH and L-902688 are cytotoxic for CLL cells MEC-1 and Burkitt lymphoma cells Ramos. MEC-1 and Ramos cells were exposed to increasing concentrations of PgE1-OH or L-902688 (i.e., 1, 5, 10, 25, 50, 100, 150 μM). The metabolic activity or viability of cells was determined after 24 h or 48 h and the IC50 values were calculated (mean ± SD of three independent experiments performed in triplicate). IC50 values represent half maximal inhibitory concentration for the metabolic activity or viability of the cells.

EP4 Receptor Agonist	Cell Line	Metabolic Activity Assay		Propidium Iodide	
		IC50 (µM) 24 h	IC50 (µM) 48 h	IC50 (µM) 24 h	IC50 (µM) 48 h
PgE1-OH	MEC-1	36.3	21.5	35.6	25.6
	Ramos	31.1	16.5	57.2	25.0
L-902688	MEC-1	7.3	5.2	7.0	5.6
	Ramos	4.0	3.5	8.4	4.7

**Table 2:** Combination indices (CI) of EP4 receptor agonists, PgE1-OH and L-902688, in combination with anti-CD20 MAbs rituximab, ofatumumab and obinutuzumab. The CIs were determined for the combination of 10 µM PgE1-OH or 5 µM L-902688 with the indicated concentrations of the anti-CD20 MAbs, using the CompuSyn software. CI ˂ 1, synergistic; CI = 1, additive; CI ˃ 1 antagonistic effect.

EP4 Receptor Agonist	Mab (µg/mL)	Concentration	CI (Ramos)	CI (MEC-1)
PgE1-OH	Rituximab	1 µg/mL	0.52	0.58
		10 µg/mL	0.46	0.58
	Ofatmumab	1 µg/mL	0.53	0.37
		10 µg/mL	0.46	0.50
	Obinutuzumab	1 µg/mL	0.97	0.10
		10 µg/mL	0.94	0.08
L-902688	Rituximab	1 µg/mL	1.17	0.85
		10 µg/mL	1.12	0.89
	Ofatmumab	1 µg/mL	1.00	0.91
		10 µg/mL	0.92	1.02
	Obinutuzumab	1 µg/mL	1.40	0.38
		10 µg/mL	1.40	0.36

## Data Availability

The data presented in this study are available on request from the corresponding author. The data are not publicly available due to privacy and ethical restrictions.

## Supplementary Materials

The following supporting information can be downloaded at: www.mdpi.com/article/10.3390/ijms23031599/s1.

## References

[1] Torre L.A., Bray F., Siegel R.L., Ferlay J., Lortet-Tieulent J., Jemal A. (2015). Global Cancer Statistics, 2012. CA Cancer J. Clin..

[2] Salles G., Barrett M., Foà R., Maurer J., O’Brien S., Valente N., Wenger M., Maloney D.G. (2017). Rituximab in B-Cell Hematologic Malignancies: A Review of 20 Years of Clinical Experience. Adv. Ther..

[3] Middleton O., Cosimo E., Dobbin E., McCaig A.M., Clarke C.L., Brant A.M., Leach M., Michie A., Wheadon H. (2015). Complement deficiencies limit CD20 monoclonal antibody treatment efficacy in CLL. Leukemia.

[4] Byrd J.C., Stilgenbauer S., Flinn I.W. (2004). Chronic Lymphocytic Leukemia. Hematology.

[5] Glennie M.J., French R.R., Cragg M.S., Taylor R.P. (2007). Mechanisms of killing by anti-CD20 monoclonal antibodies. Mol. Immunol..

[6] Hallek M. (2019). Chronic lymphocytic leukemia: 2020 update on diagnosis, risk stratification and treatment. Am. J. Hematol..

[7] Lim S.H., Beers S.A., French R.R., Johnson P.W., Glennie M.J., Cragg M.S. (2010). Anti-CD20 monoclonal antibodies: Historical and future perspectives. Haematologica.

[8] Okroj M., Österborg A., Blom A.M. (2013). Effector mechanisms of anti-CD20 monoclonal antibodies in B cell malignancies. Cancer Treat. Rev..

[9] Luan C., Chen B. (2019). Clinical application of obinutuzumab for treating chronic lymphocytic leukemia. Drug Des. Dev. Ther..

[10] McLaughlin P., Grillo-López A.J., Link B.K., Levy R., Czuczman M.S., Williams M.E., Heyman M.R., Bence-Bruckler I., White C.A., Cabanillas F. (1998). Rituximab chimeric anti-CD20 monoclonal antibody therapy for relapsed indolent lymphoma: Half of patients respond to a four-dose treatment program. J. Clin. Oncol..

[11] Davis T.A., Grillo-López A.J., White C.A., McLaughlin P., Czuczman M.S., Link B.K., Maloney D.G., Weaver R.L., Rosenberg J., Levy R. (2000). Rituximab Anti-CD20 Monoclonal Antibody Therapy in Non-Hodgkin’s Lymphoma: Safety and Efficacy of Re-Treatment. J. Clin. Oncol..

[12] Bonavida B. (2007). Rituximab-induced inhibition of antiapoptotic cell survival pathways: Implications in chemo/immunoresistance, rituximab unresponsiveness, prognostic and novel therapeutic interventions. Oncogene.

[13] Rezvani A.R., Maloney D.G. (2011). Rituximab resistance. Best Pract. Res. Clin. Haematol..

[14] Tomita A. (2016). Genetic and Epigenetic Modulation of CD20 Expression in B-Cell Malignancies: Molecular Mechanisms and Significance to Rituximab Resistance. J. Clin. Exp. Hematop..

[15] Prijatelj M., Celhar T., Gobec M., Mlinaric-Rascan I. (2012). EP4 receptor signalling in immature B cells involves cAMP and NF-κB dependent pathways. J. Pharm. Pharmacol..

[16] Prijatelj M., Celhar T., Mlinarič-Raščan I. (2011). Prostaglandin EP4 receptor enhances BCR-induced apoptosis of immature B cells. Prostaglandins Other Lipid Mediat..

[17] Markovič T., Jakopin Ž., Dolenc M.S., Mlinarič-Raščan I. (2017). Structural features of subtype-selective EP receptor modulators. Drug Discov. Today.

[18] Gobec M., Prijatelj M., Delic J., Markovic T., Mlinarič-Raščan I. (2014). Chemo-sensitizing effects of EP4 receptor-induced inactivation of nuclear factor-κB. Eur. J. Pharmacol..

[19] Nabergoj S., Markovič T., Avsec D., Gobec M., Podgornik H., Jakopin Ž., Mlinarič-Raščan I. (2021). EP4 receptor agonist L-902688 augments cytotoxic activities of ibrutinib, idelalisib, and venetoclax against chronic lymphocytic leukemia cells. Biochem. Pharmacol..

[20] Konya V., Marsche G., Schuligoi R., Heinemann A. (2013). E-type prostanoid receptor 4 (EP4) in disease and therapy. Pharmacol. Ther..

[21] Gardiner P., Copas J.L., Schneider C., Collier H. (1980). 2-Decarboxy-2-hydroxymethyl prostaglandin E1 (TR4161), a prostaglandin bronchodilator of low tracheobronchial irritancy. Prostaglandins.

[22] Nizankowska E., Sheridan A.Q., Maile M.H., Cross C.J., Niżankowski R., Prochowska K., Szczeklik A. (1985). Bronchodilatory properties of 2-decarboxy-2-hydroxymethyl prostaglandin E1. Prostaglandins.

[23] Young R.N., Billot X., Han Y., Slipetz D.A., Chauret N., Belley M., Metters K., Mathieu M.-C., Greig G.M., Denis D. (2004). Discovery and Synthesis of a Potent, Selective and Orally Bioavailable EP4 Receptor Agonist. Heterocycles.

[24] Akram A., Gibson C.L., Grubb B.D. (2013). Neuroprotection mediated by the EP4 receptor avoids the detrimental side effects of COX-2 inhibitors following ischaemic injury. Neuropharmacology.

[25] Demars K.M., McCrea A.O., Siwarski D.M., Sanz B.D., Yang C., Candelario-Jalil E. (2018). Protective Effects of L-902,688, a Prostanoid EP4 Receptor Agonist, against Acute Blood-Brain Barrier Damage in Experimental Ischemic Stroke. Front. Neurosci..

[26] Foudi N., Kotelevets L., Louedec L., Leséche G., Henin D., Chastre E., Norel X. (2008). Vasorelaxation induced by prostaglandin E2in human pulmonary vein: Role of the EP4receptor subtype. Br. J. Pharmacol..

[27] Li H.-H., Hsu H.-H., Chang G.-J., Chen I.-C., Ho W.-J., Hsu P.-C., Chen W.-J., Pang J.-H.S., Huang C.-C., Lai Y.-J. (2018). Prostanoid EP4 agonist L-902,688 activates PPARγ and attenuates pulmonary arterial hypertension. Am. J. Physiol. Cell. Mol. Physiol..

[28] Ozen G., Benyahia C., Mani S., Boukais K., Silverstein A.M., Bayles R., Nelsen A.C., Castier Y., Danel C., Mal H. (2020). Bronchodilation induced by PGE 2 is impaired in Group III pulmonary hypertension. Br. J. Pharmacol..

[29] Benyahia C., Gomez I., Kanyinda L., Boukais K., Danel C., Lesèche G., Longrois D., Norel X. (2012). PGE2 receptor (EP4) agonists: Potent dilators of human bronchi and future asthma therapy?. Pulm. Pharmacol. Ther..

[30] Murn J., Mlinaric-Rascan I., Vaigot P., Alibert O., Frouin V., Gidrol X. (2009). A Myc-regulated transcriptional network controls B-cell fate in response to BCR triggering. BMC Genom..

[31] Okroj M., Eriksson I., Österborg A., Blom A. (2013). Killing of CLL and NHL cells by rituximab and ofatumumab under limited availability of complement. Med. Oncol..

[32] Peng W., Zhang X., Mohamed N., Inghirami G., Takeshita K., Pecora A., Nardone L.L., Pincus S.E., Casey L.S., Spitalny G.L. (2005). A DeImmunized chimeric anti-C3b/iC3b monoclonal antibody enhances rituximab-mediated killing in NHL and CLL cells via complement activation. Cancer Immunol. Immunother..

[33] VanDerMeid K.R., Elliott M., Baran A., Barr P.M., Chu C.C., Zent C.S. (2018). Cellular Cytotoxicity of Next-Generation CD20 Monoclonal Antibodies. Cancer Immunol. Res..

[34] Pawluczkowycz A.W., Beurskens F.J., Beum P.V., Lindorfer M.A., Van De Winkel J.G.J., Parren P.W.H.I., Taylor R.P. (2009). Binding of Submaximal C1q Promotes Complement-Dependent Cytotoxicity (CDC) of B Cells Opsonized with Anti-CD20 mAbs Ofatumumab (OFA) or Rituximab (RTX): Considerably Higher Levels of CDC Are Induced by OFA than by RTX. J. Immunol..

[35] Hayashi K., Nagasaki E., Kan S., Ito M., Kamata Y., Homma S., Aiba K. (2016). Gemcitabine enhances rituximab-mediated complement-dependent cytotoxicity to B cell lymphoma by CD20 upregulation. Cancer Sci..

[36] Skarzynski M., Niemann C., Lee Y.S., Martyr S., Maric I., Salem D., Stetler-Stevenson M., Marti G.E., Calvo K.R., Yuan C. (2016). Interactions between Ibrutinib and Anti-CD20 Antibodies: Competing Effects on the Outcome of Combination Therapy. Clin. Cancer Res..

[37] Illidge T., Klein C., Sehn L.H., Davies A., Salles G., Cartron G. (2015). Obinutuzumab in hematologic malignancies: Lessons learned to date. Cancer Treat. Rev..

[38] Crombie J.L., Brown J.R. (2021). The future of antibody therapy in chronic lymphocytic leukemia. Expert Opin. Emerg. Drugs.

[39] Chou T.-C. (2006). Theoretical Basis, Experimental Design, and Computerized Simulation of Synergism and Antagonism in Drug Combination Studies. Pharmacol. Rev..

