# Supplementary material for: The Enhanced Cytotoxic Effects in B-Cell Leukemia and Lymphoma Following Activation of Prostaglandin EP4 Receptor and Targeting of CD20 Antigen by Monoclonal Antibodies
Source: Int J Mol Sci. 2022 Jan 29;23(3):1599. doi: 10.3390/ijms23031599 (PMC8835876; doi:10.3390/ijms23031599)
Supplement: Supplementary file 1 [file ijms-23-01599-s001.zip › ijms-1551185 suppl.pdf]

International Journal of *Molecular Sciences*

MDPI

Article

The enhanced cytotoxic effects in B cell leukemia and lymphoma following activation of prostaglandin EP4 receptor and targeting of CD20 antigen by monoclonal antibodies

Tijana Markovič [1], Helena Podgornik [1,2], Damjan Avsec [1], Sanja Nabergoj [1] and Irena Mlinarič-Raščan [1,*]

[1] University of Ljubljana, Faculty of Pharmacy, SI-1000 Ljubljana, Slovenia; tijana.markovic@ffa.uni-lj.si (T.M); damjan.avsec@ffa.uni-lj.si (D.A.); sanja.nabergoj@ffa.uni-lj.si (S.N.)
[2] University Medical Centre Ljubljana, Department of Haematology, SI-1000 Ljubljana, Slovenia; helena.podgornik@kclj.si (H.P.)
* Correspondence: irena.mlinaric-rascan@ffa.uni-lj.si

Citation: Markovič, T.; Podgornik, H.; Avsec, D.; Nabergoj, S.; Mlinarič-Raščan, I. The Enhanced Cytotoxic Effects in B-Cell Leukemia and Lymphoma Following Activation of Prostaglandin EP4 Receptor and Targeting of CD20 Antigen by Monoclonal Antibodies. *Int. J. Mol. Sci.* **2022**, *23*, 1599. https://doi.org/10.3390/ijms23031599

Academic Editors: Cinzia Solinas and Pushpamali de Silva

Received: 27 December 2021
Accepted: 27 January 2022
Published: 30 January 2022

Publisher's Note: MDPI stays neutral with regard to jurisdictional claims in published maps and institutional affiliations.

Copyright: © 2022 by the authors. Licensee MDPI, Basel, Switzerland. This article is an open access article distributed under the terms and conditions of the Creative Commons Attribution (CC BY) license (https://creativecommons.org/licenses/by/4.0/).

Supplementary information

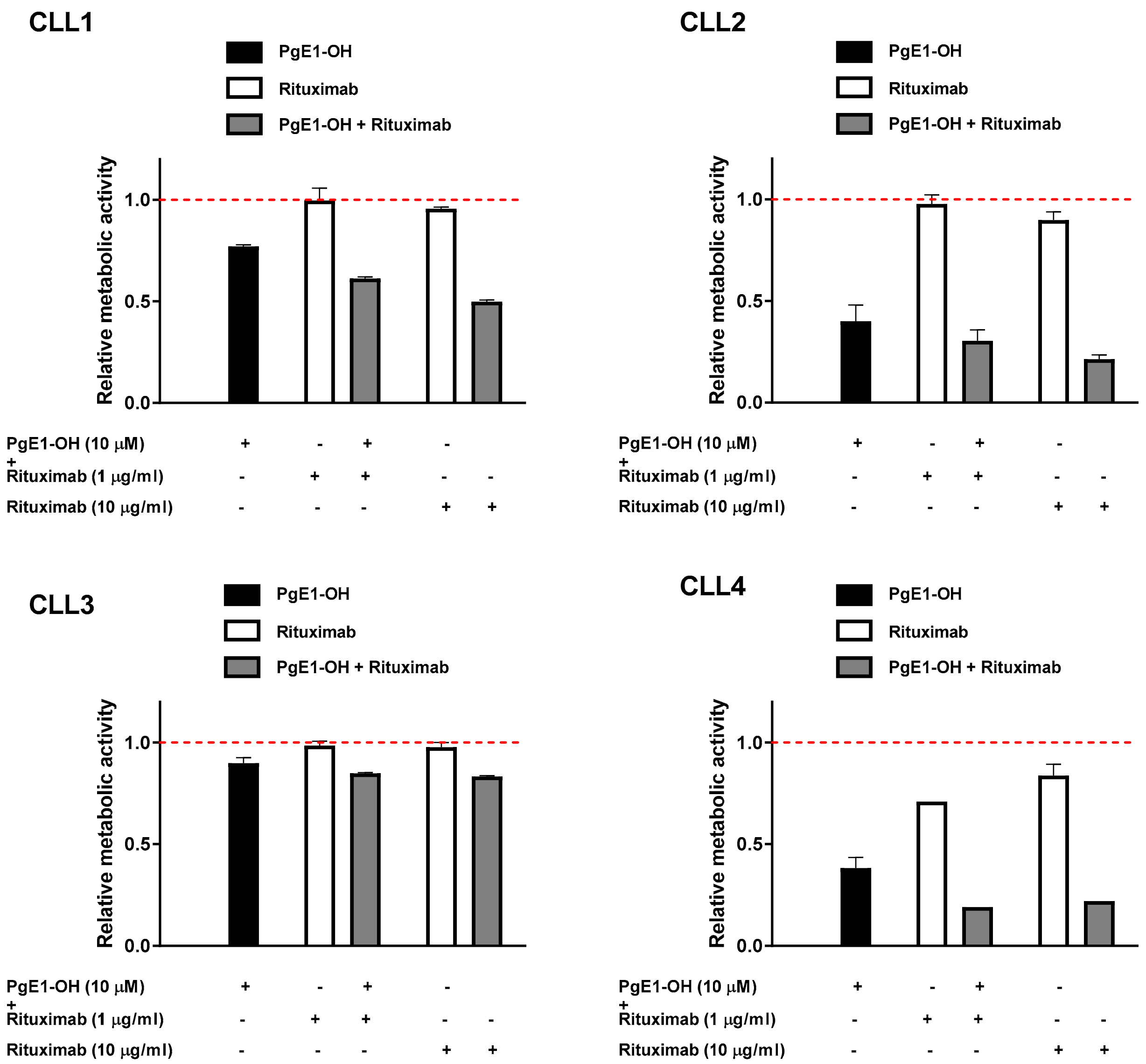

Figure S1. EP4 receptor agonists enhance the cytotoxicity of anti-CD20 Mab rituximab against primary CLL cells. Each subfigure represents one of four CLL patients. Cells were incubated with 10 μM PgE1-OH and 1 μg/ml or 10 μg/ml rituximab in the presence of one percent autologous human serum. Metabolic activity was determined after 48 h. Red dotted line represents the relative metabolic activity of untreated control cells. Data are presented as ratios relative to untreated control cells. The experiments performed in triplicate.

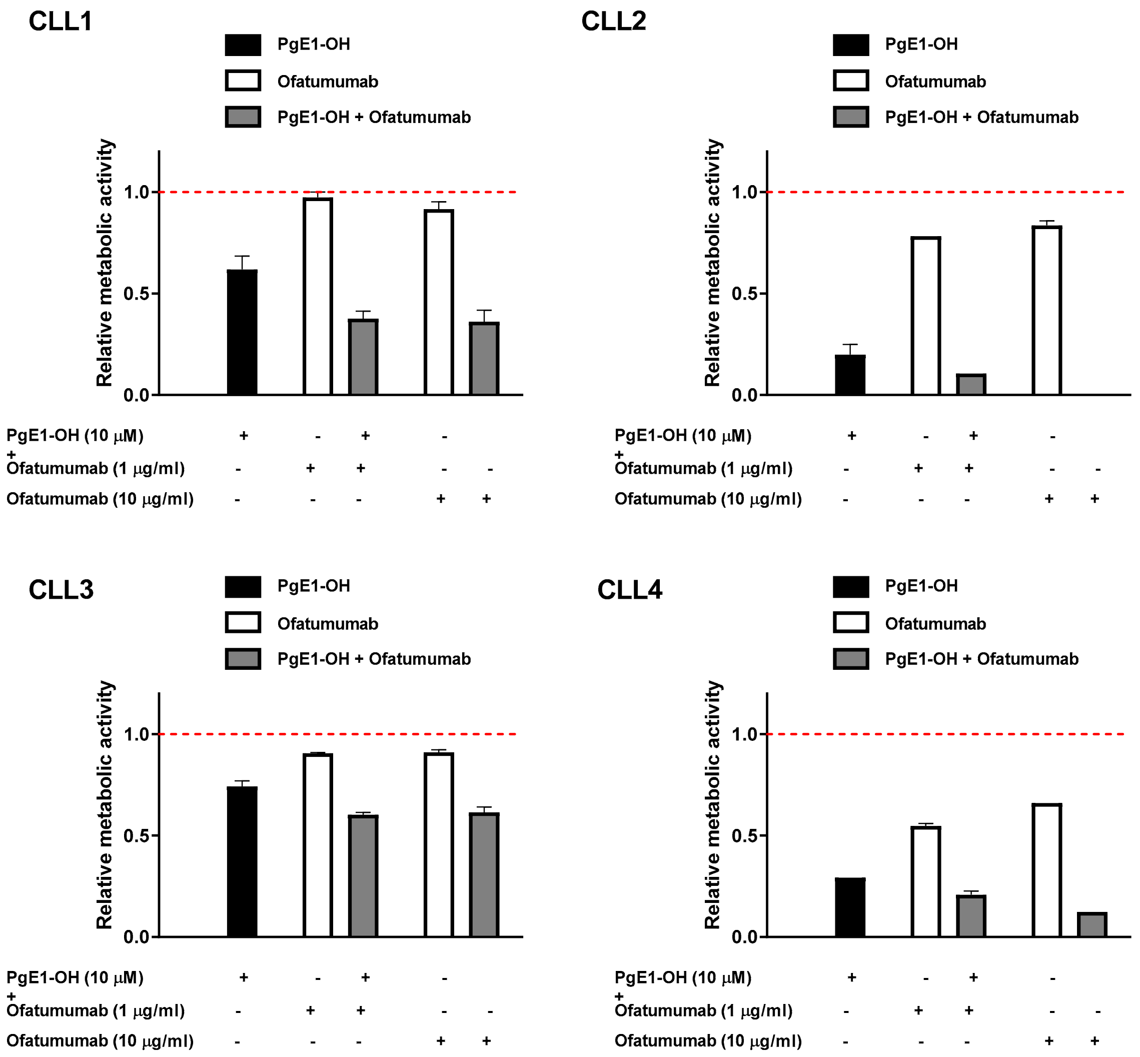

Figure S2. EP4 receptor agonists enhance the cytotoxicity of anti-CD20 Mab ofatumumab against primary CLL cells. Each subfigure represents one of four CLL patients. Cells were incubated with 10 μM PgE1-OH and 1 μg/ml or 10 μg/ml rituximab in the presence of one percent autologous human serum. Metabolic activity was determined after 48 h. Red dotted line represents the relative metabolic activity of untreated control cells. Data are presented as ratios relative to untreated control cells. The experiments performed in triplicate.
